# CX3CL1 promotes cell sensitivity to ferroptosis and is associated with the tumor microenvironment in clear cell renal cell carcinoma

**DOI:** 10.1186/s12885-022-10302-2

**Published:** 2022-11-17

**Authors:** Qiming Gong, Zhiting Guo, Wenjuan Sun, Xiuri Du, Yan Jiang, Fahui Liu

**Affiliations:** 1 Department of Nephrology, Affiliated Hospital of Youjiang Medical University for Nationalities, No.18 Zhongshan Road, Baise, 533000 Guangxi China; 2 College of Biological Science and Engineering, Fuzhou University, Fuzhou, 350108 China; 3 Department of Internal Medicine, Chonnam National University Medical School, Gwangju, 61469 South Korea; 4 Department of Rheumatology, Affiliated Hospital of Youjiang Medical University for Nationalities, No.18 Zhongshan Road, Baise, 533000 Guangxi China; 5 Science Laboratory, Youjiang Medical University for Nationalities, No.98 Chengxiang Road, Baise, 533000 Guangxi China; 6 Department of Medical Biochemistry and Cell Biology, Institute of Biomedicine, University of Gothenburg, 40530 Gothenburg, Sweden

**Keywords:** CX3CL1, Clear cell renal cell carcinoma, Tumor microenvironment, Immunotherapy, Ferroptosis

## Abstract

**Background:**

An increasing number of studies have demonstrated that CX3CL1 is involved in the development of tumors and may thus be considered a new potential therapeutic target for them. However, the function of CX3CL1 in clear cell renal cell carcinoma (ccRCC) remains poorly defined.

**Methods:**

The pan-cancer expression pattern and prognostic value of CX3CL1 were evaluated in this study. Moreover, the relationship of CX3CL1 expression with the tumor microenvironment, especially the tumor immune microenvironment, was analyzed. Our analyses employed public repository data. Additionally, we generated stable CX3CL1-overexpressing 786-O cells to determine the role of CX3CL1 in *vitro* via cell viability and transwell assays. A xenograft tumor model was used to determine the role of CX3CL1 in *vivo*. The association between CX3CL1 and ferroptosis sensitivity of tumor cells was assessed using Ferrostatin-1.

**Results:**

Our findings indicated the involvement of CX3CL1 in the occurrence and development of ccRCC by acting as a tumor suppressor. We also found that ccRCC patients with high CX3CL1 expression showed better clinical outcomes than those with low CX3CL1 expression. The findings of our epigenetic study suggested that the expression of CX3CL1 in ccRCC is correlated with its DNA methylation level. Furthermore, the CX3CL1 expression level was closely related to the infiltration level of CD8^+^ T cells into the tumor microenvironment (TME). CX3CL1 showed different predictive values in different immunotherapy cohorts. Finally, CX3CL1 overexpression inhibited tumor cell proliferation and metastasis and promoted tumor ferroptosis sensitivity in ccRCC.

**Conclusions:**

This study revealed the role of CX3CL1 as a tumor suppressor in ccRCC. Our findings indicated that CX3CL1 plays a crucial role in regulating the ccRCC TME and is a potential predictor of immunotherapy outcomes in ccRCC. We also found that CX3CL1 can promote ferroptosis sensitivity in ccRCC cells.

**Supplementary Information:**

The online version contains supplementary material available at 10.1186/s12885-022-10302-2.

## Introduction

Clear cell renal cell carcinoma (ccRCC), a kidney malignancy, accounts for about 80% of renal cell carcinoma (RCC) cases [1]. Most patients with ccRCC are diagnosed at an advanced stage of the disease due to its asymptomatic nature and the absence of screening methods for early-stage detection. The use of immune checkpoint inhibitors and targeted therapies, such as tyrosine kinase inhibitors, has partially improved the prognosis of patients with ccRCC [2]. However, more effective therapeutic modalities are required to prolong the survival of patients with ccRCC. Recent studies have reported that ferroptosis is inhibited in tumor cells through metabolic reprogramming in ccRCC [3]. Thus, ferroptosis induction in tumor cells is a potential therapeutic strategy for ccRCC. Ferroptosis is a type of regulated cell death that is mediated by iron and lipid reactive oxygen species and results in a “ballooning” phenotype in some cell types [4–6]. Intriguingly, ferroptosis has been correlated with antitumor immunity in numerous cancers [7]. The sensitivity of cancer cells to ferroptosis thus affects immunogenic cell death and immunotherapy outcomes [3]. However, the relationship between immunotherapy and ferroptosis in ccRCC still needs to be elucidated.

Up to now, approximately 50 chemokines have been discovered [8]. The chemokine CX3CL1 and its receptor CX3CR1 play a complex role in various human tumors’ development, progression, and metastasis [9, 10]. Previous studies reported the pro-tumor effects of CX3CL1-CX3CR1 signaling in pancreatic, breast, lung, and prostate cancers through activating the EGFR-Src FAK axis among others [11–15]. Studies have reported that CX3CL1 may exert anti-tumor effects in colorectal cancer and glioma via anticancer T cells and NK cells [16, 17]. Despite recent progress, the molecular mechanisms underlying ccRCC are still elusive, and the role and prognostic value of CX3CL1 in ccRCC have not been reported.

In this study, we focused on exploring the role of CX3CL1 in ccRCC through a comprehensive multi-omics analysis combined with molecular biology experiments. Our findings demonstrate the role of CX3CL1 in regulating the tumor immune microenvironment and its potential as a clinical prognostic marker for predicting the efficacy of immunotherapy. In addition, the function of CX3CL1 as a ferroptosis sensitivity regulator in ccRCC was confirmed by molecular biology experiments. Overall, these findings suggest that CX3CL1 can act as a therapeutic target in ccRCC by affecting ferroptosis and the tumor immune microenvironment.

## Materials and methods

### Data download

All RNAseq data of The Cancer Genome Atlas (TCGA) and Genotype-Tissue Expression (GTEx) in TPM format processed by the Toil process were downloaded from UCSC Xena (https://xenabrowser.net/datapages/). Log2 (TPM + 1) transformed values were used for the analyses. A total of 33 types of cancers were included in this study. The full name of each cancer type and abbreviations are provided in Supplementary Table 1. The expression data of ENSG00000006210 (CX3CL1) were extracted for each sample. Survival information for patients in TCGA, including overall survival, disease-free survival, disease-specific survival, and progression-free survival data, was obtained from a prognostic study in TCGA [18]. The 450 K methylation data for patients with ccRCC in TCGA were downloaded from UCSC Xena (https://xenabrowser.net/datapages/). scRNA seq-data GSE121636 was downloaded from GEO database.

### Bioinformatics analysis

For the DNA methylation analysis, the methylation level for CX3CL1 in ccRCC patients was expressed as a beta value (from 0 = unmethylated to 1 = fully methylated). For the survival analysis of CX3CL1 in pan-cancer, the cox regression model was established to analyze the relationship between CX3CL1 gene expression and different clinical outcomes in each tumor. A log-rank test was used to assess statistical significance. For survival analysis of CX3CL1 in ccRCC, patients were divided into high and low-expression groups according to the median value of CX3CL1 (including the expression level of CX3CL1 and the methylation level of each CX3CL1 methylation site). Survival was assessed using the Kaplan-Meier method, and a log-rank test was used for statistical analysis. Univariate and multivariate analyses were used to identify independent prognostic factors in ccRCC. Biomarker Exploration of Solid Tumors (BEST) web server (https://rookieutopia.com/) was used to analyze the correlations between CX3CL1 expression and immune cells infiltration in different cohorts using multiple algorithms. The expression level of CX3CL1 in different immunotherapy cohorts and the relationship between CX3CL1 expression and the prognosis of patients given immunotherapy were calculated using the BEST web server.

### Establishment of ccRCC models in nude mice

All nude mice (male, 22.0 ± 1.2 g) were purchased from shanghai and maintained at Youjiang Medical University for Nationalities’ SPF facility (NO. SYXK 2017-0004). 786-O cells stably transfected with normal vector and CX3CL1-overexpressing (1 × 10^6^) were injected into the right flank of nude mice (5 mice/group). Tumors were detected once every 3 days. The formula tumor volume = a × b^2^/2 (where a represents the largest tumor diameters; b represents the smallest tumor diameters). Eight weeks after injection, tumor tissues were extracted and processed for Western Blot and Immunofluorescence assay. All surgeries were carried out under pentobarbital anesthesia to alleviate mouse suffering. All procedures were performed in accordance with animal experimentation ethics at Youjiang Medical University for Nationalities.

### Cell culture and transfection

The human ccRCC cell line 786-O was obtained from the Chinese Academy of Sciences. 786-O cells were incubated with Ferrostatin-1 (Fer-1, S7243, Selleckchem, Houston, TX, USA), Necrostatin-1 (MCE, HY-15760), and Z-VAD-FMK (MCE, HY-16658B). 786-O cells were infected with small interfering RNA (Si-RNA) (Ubi-MCS-CBh-gcGFP-IRES-Puro-CX3CL1) against CX3CL1 and infected with a lentiviral vector (Ubi-MCS-3FLAG-SV40-mcherry-IRES-Puro-CX3CL1) (Shanghai Gene Chem Co., Ltd.) to induce CX3CL1 overexpression.

### Cells viability assay

786-O cells were seeded into 96-well plates at 3× 10^3^ cells per well. Cells were incubated with Fer-1 (0, 1, 2, and 4 μM/ml) for 48, 72, 96 and 120 h. Cell Counting Kit-8 kit (M4839, AbMole, Beijing, China) was used to estimate cell viability, and OD 450 nm assays were performed using the TriStar LB 941 multimode microplate reader (Berthold Technologies, Germany).

### Transwell assay

Transwell experiments were performed using an 8 μm transwell chamber (corning, USA). Briefly, 786-O cells were plated onto the upper chamber pre-coated with Matrigel (corning, USA). The complete medium was filled in the lower chamber. After incubation, 786-O cells were fixed with paraformaldehyde and stained with 0.1% crystal violet solution. Samples numbers were acquired using a microscope.

### Colony formation assay

All cells for colony formation assay were digested and seeded directly in 6-well plates (5 × 10^2^ cells/well) and cultured at 37 °C with 5% CO2. After 18 days, the medium was removed, and the wells were washed with phosphate-buffered saline (PBS) three times at room temperature. The cells were fixed with paraformaldehyde (1 ml/well) for 30 minutes and then stained with crystal violet solution (1 ml/well) for 30 minutes.

### Western blotting and immunofluorescence assay

For western blotting, protein samples obtained from tissues and cellular extracts were incubated with anti-CX3CL1 (Cat# DF12376, Affinity Biosciences), anti-PCNA (Cat# AF0239, Affinity Biosciences), anti-GPX4 (Cat#DF6701, Affinity Biosciences), and anti-XCT (Cat# DF12509, Affinity Biosciences) antibodies. For the immunofluorescence assay, tissues and cells were fixed, their membrane was broken, and they were sealed in slides; they were then incubated with anti-CX3CL1, anti-PCNA, anti-GPX4, and anti-XCT antibodies. Specific details of these experiments have been described previously [19].

### Immunohistochemistry (IHC) assay

Mice tissue sections (5 μm) were permeabilized with Triton X-100 in PBS, then blocked with 10% goat serum, and incubated with anti-CX3CL1, anti-GPX4, and anti-PCNA antibodies at 4 °C overnight. Then the sections were incubated with HRP-conjugated goat anti-rabbit IgG secondary antibodies (Cat#S0001, Affinity Biosciences). After counterstaining, sections were dehydrated and imaged using a microscope.

### Statistical analysis

Spearman’s rank correlation was used to assess the association between two continuous variables. Statistical significance was estimated using unpaired Student’s t-test for normally distributed variables and the Mann-Whitney U test for non-normally distributed variables. The Kruskal-Wallis test was used to compare more than two groups. All statistical analyses were done with R (version 4.1.0, www.r-project.org) and GraphPad Prism 9.

## Results

### Function of CX3CL1

**Fig. 1:**
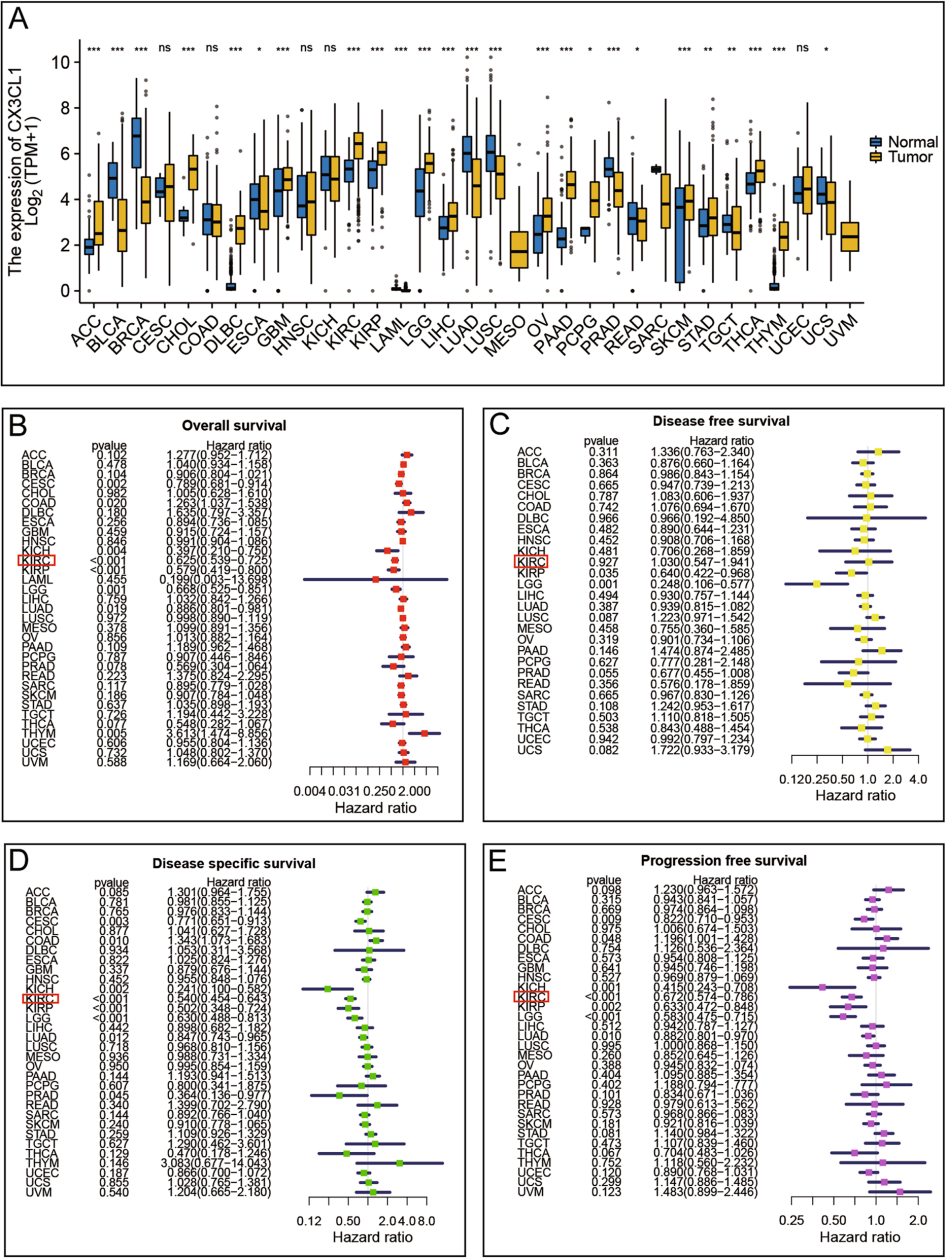
Expression of CX3CL1 in different cancers and prognostic values. **A** CX3CL1 expression in different cancers in the TCGA database. **B-E** Forest plot prognosis HR of CX3CL1 in various cancers in terms of overall survival, disease-free survival, disease-specific survival, and progression-free survival

TCGA and GTEx data from the UCSC Xena database were employed to evaluate the expression levels of CX3CL1 in cancer tissues compared with those in normal tissues. The pan-cancer analysis results showed that the expression of CX3CL1 was higher in the tissues of 15 types of cancers, including ACC, CHOL, DLBC, GBM, KIRC, KIRP, LGG, LIHC, OV, PAAD, PCPG, SKCM, STAD, THCA, and THYM, compared to that in normal tissues. The expression levels of CX3CL1 in tissues of BLCA, BRCA, ESCA, LAML, LUAD, LUSC, PRAD, READ, TGCT, and UCS cancers were lower than those in normal tissues (Fig. 1A). Next, we explored the correlation between survival data and CX3CL1 expression. In the overall survival (OS) analysis, higher CX3CL1 expression was associated with worse prognosis in COAD (HR = 1.263, *P* = 0.020) and THYM (HR = 3.613, *P* = 0.005), whereas, lower CX3CL1 expression was associated with worse prognosis in CESC (HR = 0.789, *P* = 0.002), KICH (HR = 0.397, *P* = 0.004), KIRC (HR = 0.625, *P* <  0.001), KIRP (HR = 0.579, *P* <  0.001), LGG (HR = 0.668, *P* = 0.001), LUAD (HR = 0.886, *P* = 0.019) (Fig. 1B). Analysis of disease-free survival (DFS) data revealed that lower CX3CL1 expression was associated with worse prognosis in KIRP (HR = 0.640, *P* = 0.035) and LGG (HR = 0.248, *P* = 0.001) (Fig. 1C). Next, we performed a disease-specific survival (DSS) analysis, which showed that higher CX3CL1 expression was associated with worse prognosis in COAD (HR = 1.343, *P* = 0.010) and lower CX3CL1 expression was associated with worse prognosis in CESC (HR = 0.771, *P* = 0.003), KICH (HR = 0.241, *P* = 0.002), KIRC (HR = 0.540, *P* <  0.001), KIRP (HR = 0.502, *P* <  0.001), LGG (HR = 0.630, *P* <  0.001), LUAD (HR = 0.847, *P* = 0.012) and PRAD (HR = 0.364, *P* = 0.045) (Fig. 1D). Among these data, those for KIRC (KIRC is another name for clear cell renal cell carcinoma) piqued our interest; however, the function of CX3CL1 in clear cell renal cell carcinoma (ccRCC) remained unclear. Progression-free survival (PFS) analysis further indicated that lower CX3CL1 expression was closely associated with worse prognosis in KIRC (HR = 0.672, *P* <  0.001) (Fig. 1E). Thus, we focused on the association between ccRCC and CX3CL1 in the present study. Overall, the abovementioned results demonstrate the important role of CX3CL1 as a tumor regulatory gene in human tumors and highlight the potential of CX3CL1 as a prognostic target in ccRCC.

### Association between CX3CL1 expression and ccRCC

**Fig. 2:**
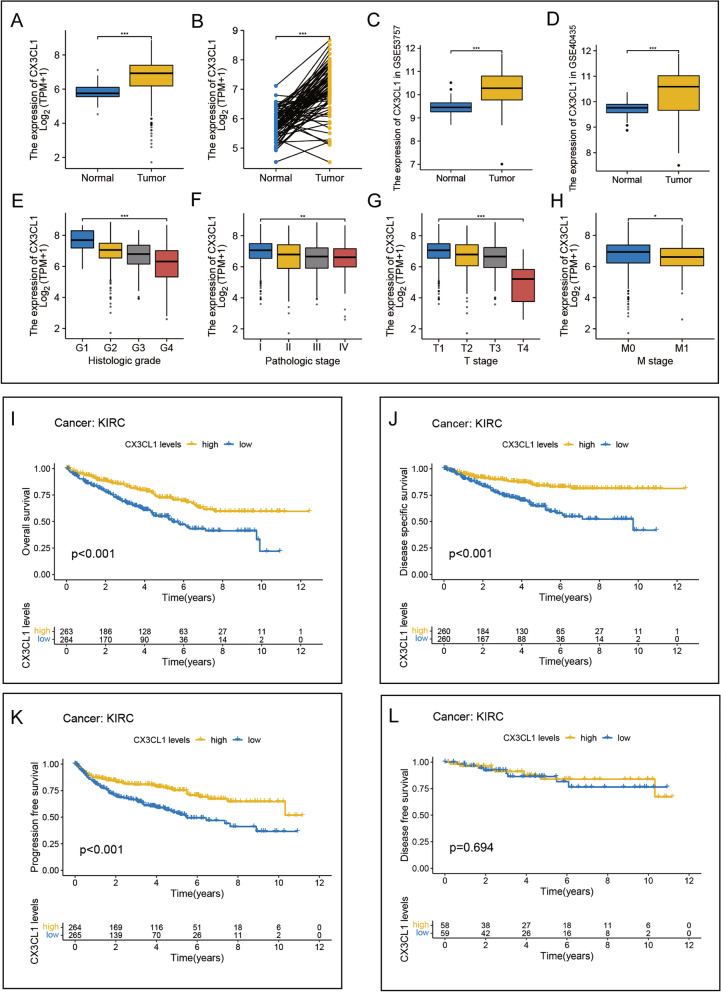
Expression of CX3CL1 in ccRCC and prognostic values. **A, B** CX3CL1 expression was higher in ccRCC tumor samples than in normal tissues in TCGA data (*P* < 0.001). **C** High expression of CX3CL1 in ccRCC in GSE53757 (*P* < 0.001). **D** High expression of CX3CL1 in ccRCC in GSE40435 (*P* < 0.001). **E-H** CX3CL1 is differentially expressed in different histologic grade, pathologic stage, T stage, and M stage groups (*P* < 0.05). *P < 0.05, **P < 0.01, ***P < 0.001. **I-L** The relationship between the expression of CX3CL1 and overall survival, disease-free survival, disease-specific survival, and progression-free survival in ccRCC

Compared with that in normal tissues, the mRNA expression level of CX3CL1 in TCGA-KIRC tissues was upregulated. Paired analysis of samples showed that the expression levels of CX3CL1 in tumor tissues were significantly higher than those in the paired normal tissues (Fig. 2A–B). The microarray data of patients with ccRCC from the GSE53757 (*P* <  0.001) and GSE40435 (*P* <  0.001) cohorts further validated the CX3CL1 expression difference between tumor and non-tumor tissue samples (Fig. 2C–D); the expression of CX3CL1 was significantly higher in tumor tissues than in normal tissues. Table 1 shows the different baseline characteristics of the low- and high-CX3CL1 expression groups in the TCGA cohort.

**Table 1:** Clinical information of ccRCC patients

Characteristic	Low expression of CX3CL1	High expression of CX3CL1	*P* value
n	269	270	
T stage, n (%)			**< 0.001**
T1	113 (21%)	165 (30.6%)	
T2	39 (7.2%)	32 (5.9%)	
T3	107 (19.9%)	72 (13.4%)	
T4	10 (1.9%)	1 (0.2%)	
N stage, n (%)			0.132
N0	126 (49%)	115 (44.7%)	
N1	12 (4.7%)	4 (1.6%)	
M stage, n (%)			**0.042**
M0	212 (41.9%)	216 (42.7%)	
M1	49 (9.7%)	29 (5.7%)	
Pathologic stage, n (%)			**< 0.001**
Stage I	110 (20.5%)	162 (30.2%)	
Stage II	31 (5.8%)	28 (5.2%)	
Stage III	74 (13.8%)	49 (9.1%)	
Stage IV	52 (9.7%)	30 (5.6%)	
Primary therapy outcome, n (%)			**0.010**
PD	8 (5.4%)	3 (2%)	
SD	0 (0%)	6 (4.1%)	
PR	0 (0%)	2 (1.4%)	
CR	49 (33.3%)	79 (53.7%)	
Histologic grade, n (%)			**< 0.001**
G1	1 (0.2%)	13 (2.4%)	
G2	94 (17.7%)	141 (26.6%)	
G3	116 (21.8%)	91 (17.1%)	
G4	52 (9.8%)	23 (4.3%)	
Hemoglobin, n (%)			**0.002**
Elevated	1 (0.2%)	4 (0.9%)	
Low	152 (33.1%)	111 (24.2%)	
Normal	82 (17.9%)	109 (23.7%)	

To further understand the clinical significance of CX3CL1 in ccRCC, the differences in CX3CL1 expression among tissues with different clinicopathological features were analyzed. The results showed significant differences in CX3CL1 expression among different subgroups of histologic grade (*P* <  0.001), T stage (*P* <  0.001), M stage (*P* <  0.05), and pathological stage (*P* <  0.001), suggesting that the higher the expression level of CX3CL1, the lower is the tumor malignancy in ccRCC patients (Fig. 2E–H). Finally, survival analysis of CX3CL1 in ccRCC was performed in terms of OS (*P* <  0.001), DSS (*P* <  0.001), PFS (*P* <  0.001), and DFS (*P* = 0.694) (Fig. 2I–L). High expression of CX3CL1 in ccRCC was associated with a better patient prognosis. Uni- and multivariate analyses revealed that CX3CL1 acts as an independent favorable prognostic factor for ccRCC (*P* <  0.001) (Table 2). These results indicate the potential of CX3CL1 as a clinical prognostic marker for ccRCC.

**Table 2:** Univariate and multivariate Cox regression analyses

Characteristics	Total(N)	Univariate analysis		Multivariate analysis	
		Hazard ratio (95% CI)	*P* value	Hazard ratio (95% CI)	*P* value
T stage	539				
T1&T2	349	Reference			
T3&T4	190	3.228 (2.382–4.374)	**< 0.001**	2.055 (1.300-3.250)	**0.002**
N stage	257				
N0	241	Reference			
N1	16	3.453 (1.832–6.508)	**< 0.001**	1.602 (0.815–3.148)	0.171
M stage	506				
M0	428	Reference			
M1	78	4.389 (3.212–5.999)	**< 0.001**	3.089 (1.922–4.963)	**< 0.001**
Gender	539				
Female	186	Reference			
Male	353	0.930 (0.682–1.268)	0.648		
Age	539				
≤ 60	269	Reference			
> 60	270	1.765 (1.298–2.398)	**< 0.001**	1.858 (1.216–2.840)	**0.004**
CX3CL1	539	0.688 (0.609–0.776)	**< 0.001**	0.682 (0.562–0.826)	**< 0.001**

### Regulation of CX3CL1 expression by DNA methylation

**Fig. 3:**
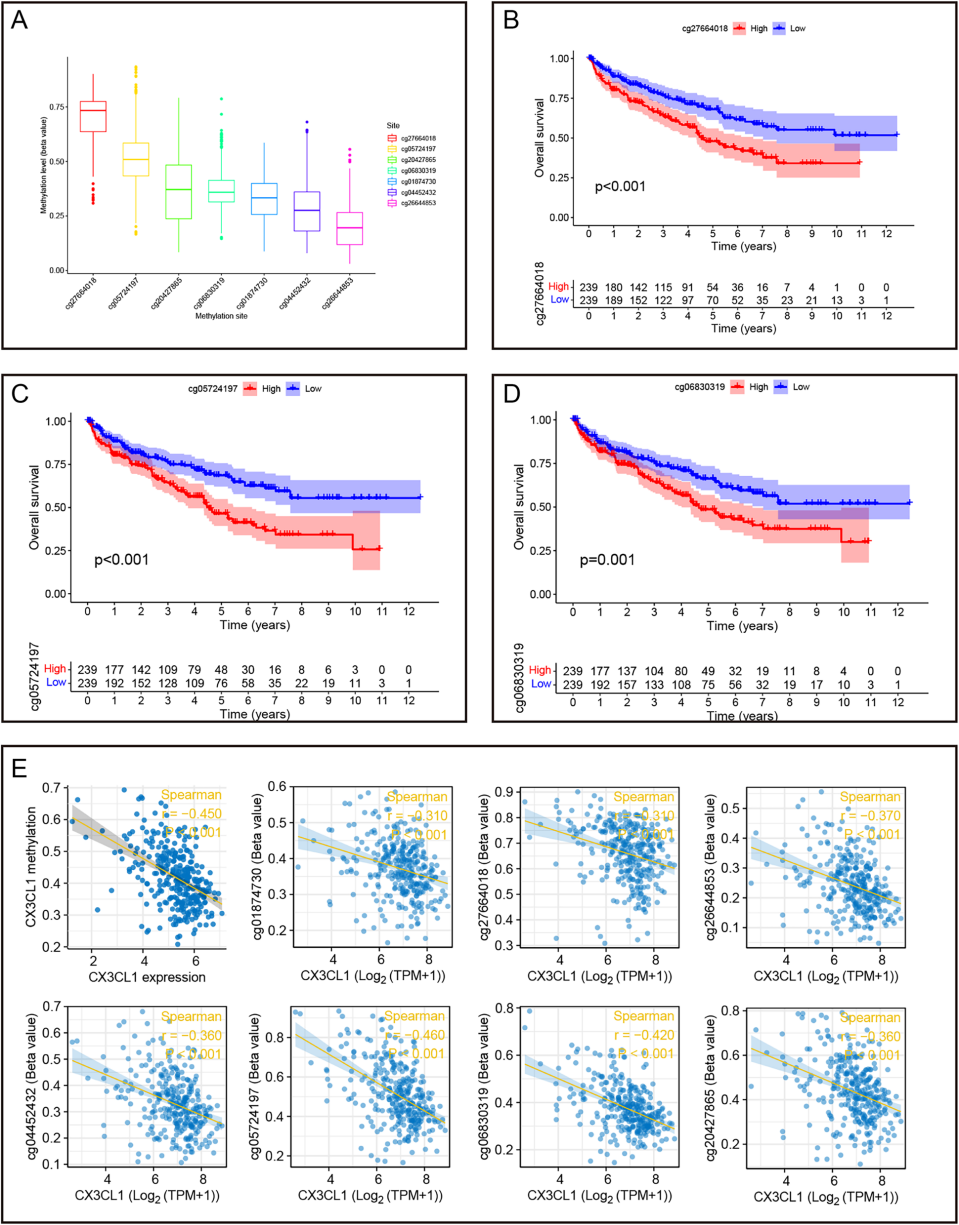
The CX3CL1 methylation levels in ccRCC from TCGA data. **A** The methylation levels of seven methylation sites on *CX3CL1* in ccRCC. **B-D** The expression of CX3CL1 was significantly associated with overall survival rates of patients with ccRCC at cg27664018 (*P* < 0.001) and cg05724197 (*P* < 0.001), cg06830319 (*P* = 0.001) methylation sites. **E** The expression of CX3CL1 was associated with CX3CL1 methylation level (*P* < 0.001)

DNA methylation plays a critical role in tumor development through several processes. We, therefore, investigated the role of DNA methylation in regulating the expression level of CX3CL1. We identified seven methylation sites in CX3CL1. The highest methylation level was detected at cg27664018, while the lowest was at cg26644853 (Fig. 3A). Next, the relationships between the DNA methylation levels at different sites of CX3CL1 and the prognosis of patients with ccRCC were analyzed. The methylation levels of cg27664018, cg05724197, and cg06830319 were negatively correlated with the prognosis of patients with ccRCC. Patients with ccRCC who had higher methylation levels of cg27664018 (*P* <  0.001), cg05724197 (*P* <  0.001), and cg06830319 (*P* = 0.001) showed a worse prognosis (Fig. 3B–D). Furthermore, a correlation analysis between the DNA methylation level and gene expression of CX3CL1 was performed. The results showed that the CX3CL1 methylation level was negatively correlated with the CX3CL1 expression level in ccRCC (*r* = − 0.405, *P* <  0.001). Further analysis of each methylation site showed that cg01874730 (*r* = − 0.310, *P* <  0.001), cg04452432 (*r* = − 0.360, *P* < 0.001), cg05724197 (*r* = − 0.460, *P* < 0.001), cg06830319 (*r* = − 0.420, *P* < 0.001), cg20427865 (*r* = − 0.360, *P* < 0.001), cg26644853 (*r* = − 0.370, *P* < 0.001), and cg27664018 (*r* = − 0.310, *P* < 0.001) were all significantly negatively correlated with the expression of CX3CL1 (Fig. 3E). These results suggest that CX3CL1 DNA methylation may influence ccRCC tumorigenesis, tumor progression, and patient prognosis.

### Immune cell infiltration analyses in ccRCC

**Fig. 4:**
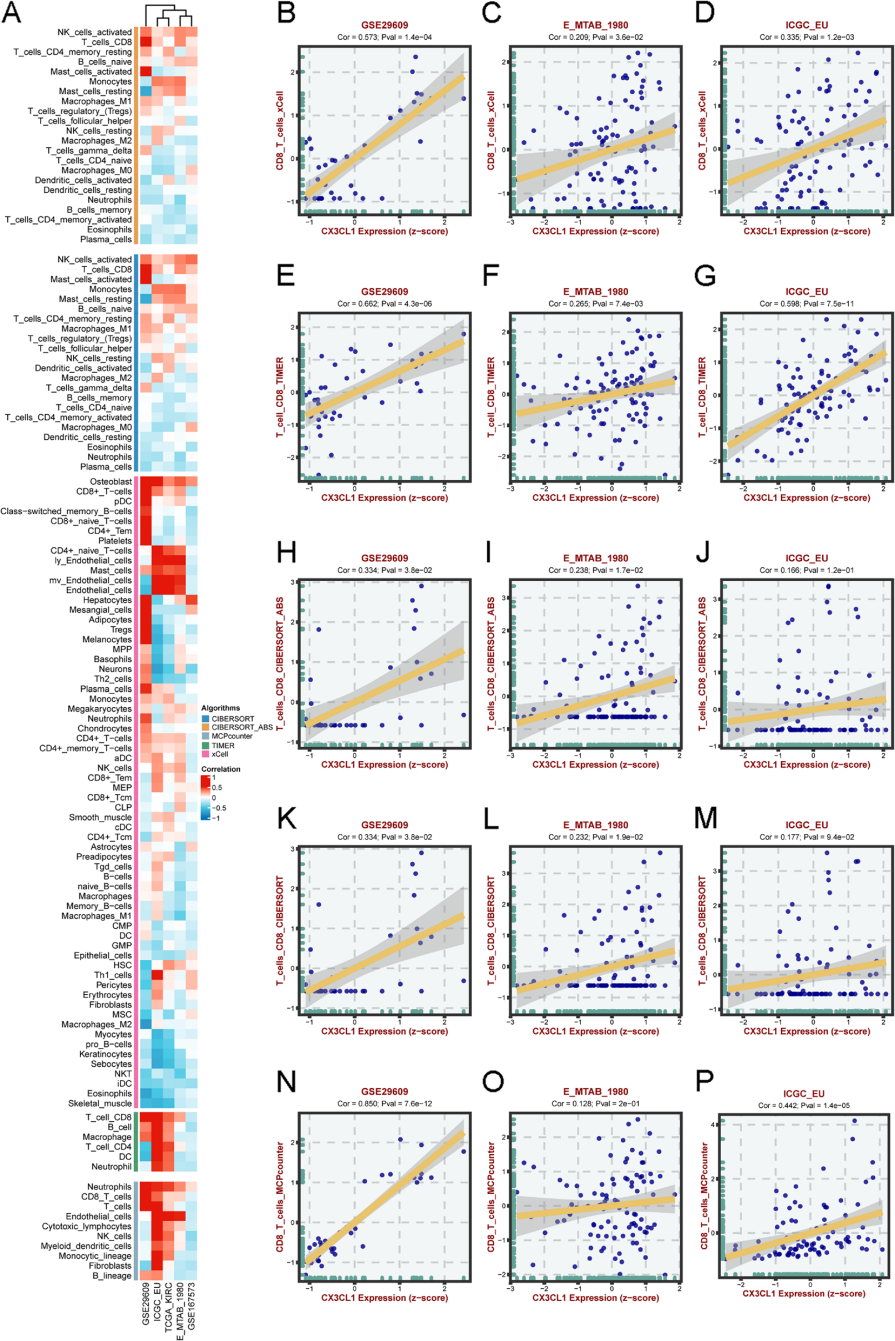
Correlation analysis of CX3CL1 expression and immune cell infiltration levels in different databases. **A** Correlation analysis between CX3CL1 expression and infiltration levels of different immune cells in GSE29609, ICGC_EU, TCGA-KIRC, E_MTAB_1980, and GSE167573 cohorts by different algorithms, including CIBERSORT, CIBERSORT_ABS, MCPcounter, TIMER, and xCell. **B-D** The association between CX3CL1 expression and CD8^+^ T cells in GSE29609, E_MTAB_1980, and ICGC_EU, was analyzed by xCell. **E-G** The association between CX3CL1 expression and CD8^+^ T cells in GSE29609, E_MTAB_1980, and ICGC_EU, was analyzed by TIMER. **H-J** The association between CX3CL1 expression and CD8^+^ T cells in GSE29609, E_MTAB_1980, and ICGC_EU was analyzed by CIBERSORT_ABS. **K-M** The association between CX3CL1 expression and CD8^+^ T cells in GSE29609, E_MTAB_1980, and ICGC_EU, was analyzed by CIBERSORT. **N-P** The association between CX3CL1 expression and CD8^+^ T cells in GSE29609, E_MTAB_1980, and ICGC_EU, was analyzed by MCPcounter

Chemokines are critical immune regulators in the tumor microenvironment (TME). We aimed to analyze cancer immunity regulation by CX3CL1; therefore, we explored the correlation between CX3CL1 expression and immune cell infiltration into the TME. Spearman correlation analyses were conducted using ccRCC immune cell infiltration data from the BEST web server (Fig. 4A). We found that CX3CL1 was positively correlated with the infiltration levels of activated CD8^+^ T cells, mast cells, and activated NK cells and negatively correlated with the infiltration levels of Th2 cells, Treg cells, and macrophages. We then evaluated the relationship between CD8^+^ T cells and CX3CL1 expression in various cohorts, including GSE29609, E_MTAB_1980, and ICG_EU, using the following four algorithms: xCell, TIMER, CIBERSORT_ABS, CIBERSORT, and MCPcounter (Fig. 4B–P). The evaluation results showed that the CX3CL1 expression level was closely related to CD8^+^ T cell infiltration into the TME. We also analyzed the relationship between TME infiltration by activated NK cells and the CX3CL1 expression level using CIBERSORT and CIBERSORT_ABS (Supplementary Fig. 1A–F). We found that the correlations of the CX3CL1 expression level with different differentiation statuses of a single cell type were different. For example, CIBERSORT analysis showed that the extent of correlation between the CX3CL1 expression level and the macropahges_M0 infiltration into the TME was different from the TME infiltration levels of macropahges_M1 and macropahges_M2. Studies have indicated that CD8^+^ T cells, NK cells, and neutrophils, among other immune cells, play a crucial role in tumor immunotherapy. The abovementioned results indicate that CX3CL1 might affect the development, prognosis, and treatment of ccRCC by influencing immune cell infiltration into the TME.

### Single-cell analysis of CX3CL1 in ccRCC

**Fig. 5:**
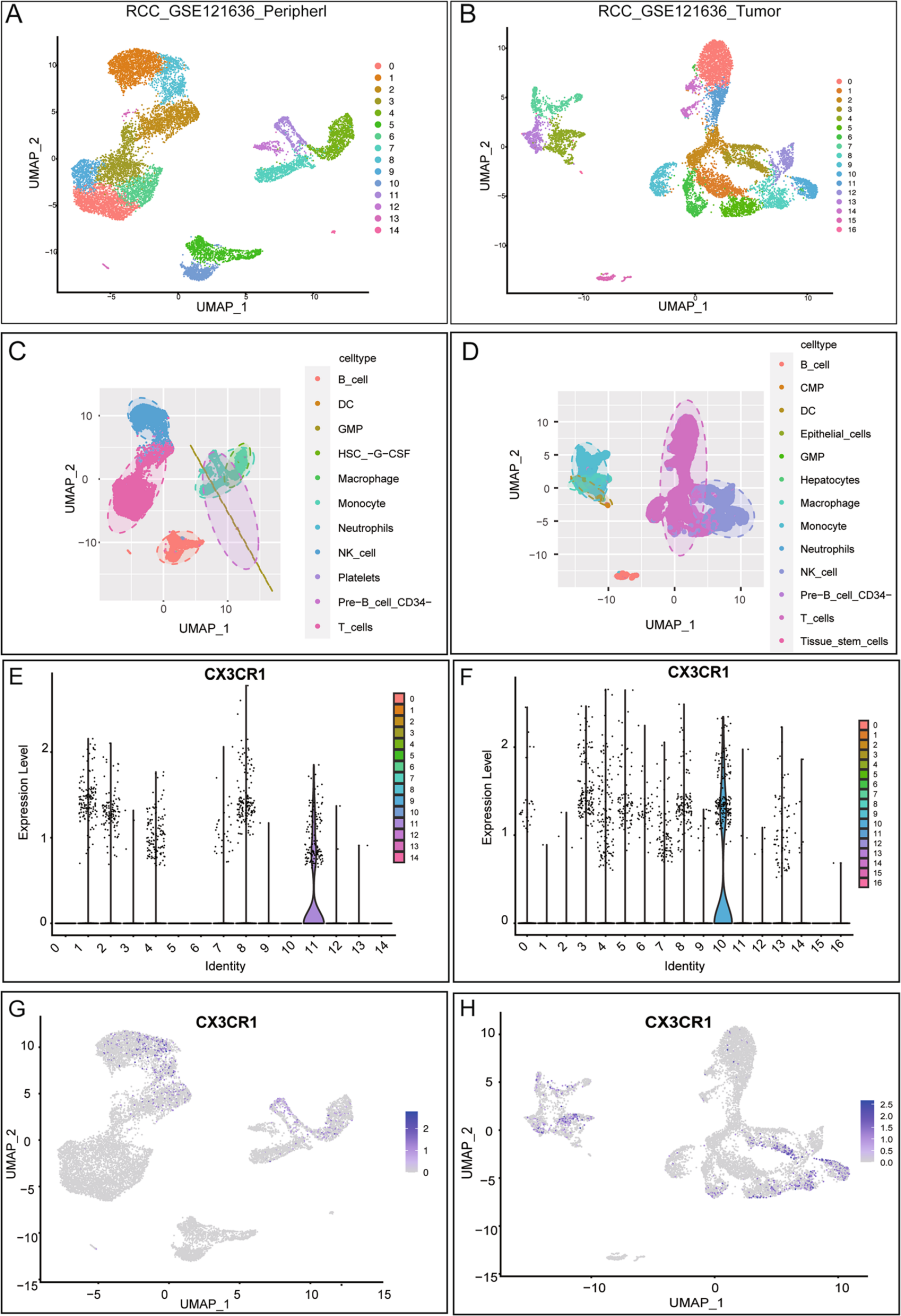
Single-cell atlas of ccRCC tissues. **A-B** UMAP of peripheral blood and tumor in ccRCC of GSE121636. **C-D** UMAP demonstrates different cell types in peripheral blood and tumor by singleR. **E-F** The relationship between the expression of CX3CR1 in different groups in peripheral blood and tumor. **G-H** The relationship between the expression of CX3CR1 in different cell types in peripheral blood and tumor

To determine the main cell types that express CX3CL1 in the ccRCC TME, we performed a single-cell analysis using the GSE121636 dataset. We determined that 15 cell types expressed CX3CL1 in peripheral blood, and 17 expressed it in the tumor (Fig. 5A–B). We then used SingleR to identify the cell types expressing CX3CLR1, the receptor of CX3CL1, in the tumor and peripheral blood (Fig. 5C–D). We found that CX3CR1 was highly expressed in the tumor and peripheral blood in group 11 and group 10, respectively. This indicated that the expression of CX3CR1 might differ between different microenvironments (Fig. 5E–F). Additionally, after annotation, most cells in groups 10 and 11 were found to be NK and T cells, corroborating our previous findings indicating that CX3CL1 is highly correlated with T cells.

### Association of CX3CL1 with immunotherapy

**Fig. 6:**
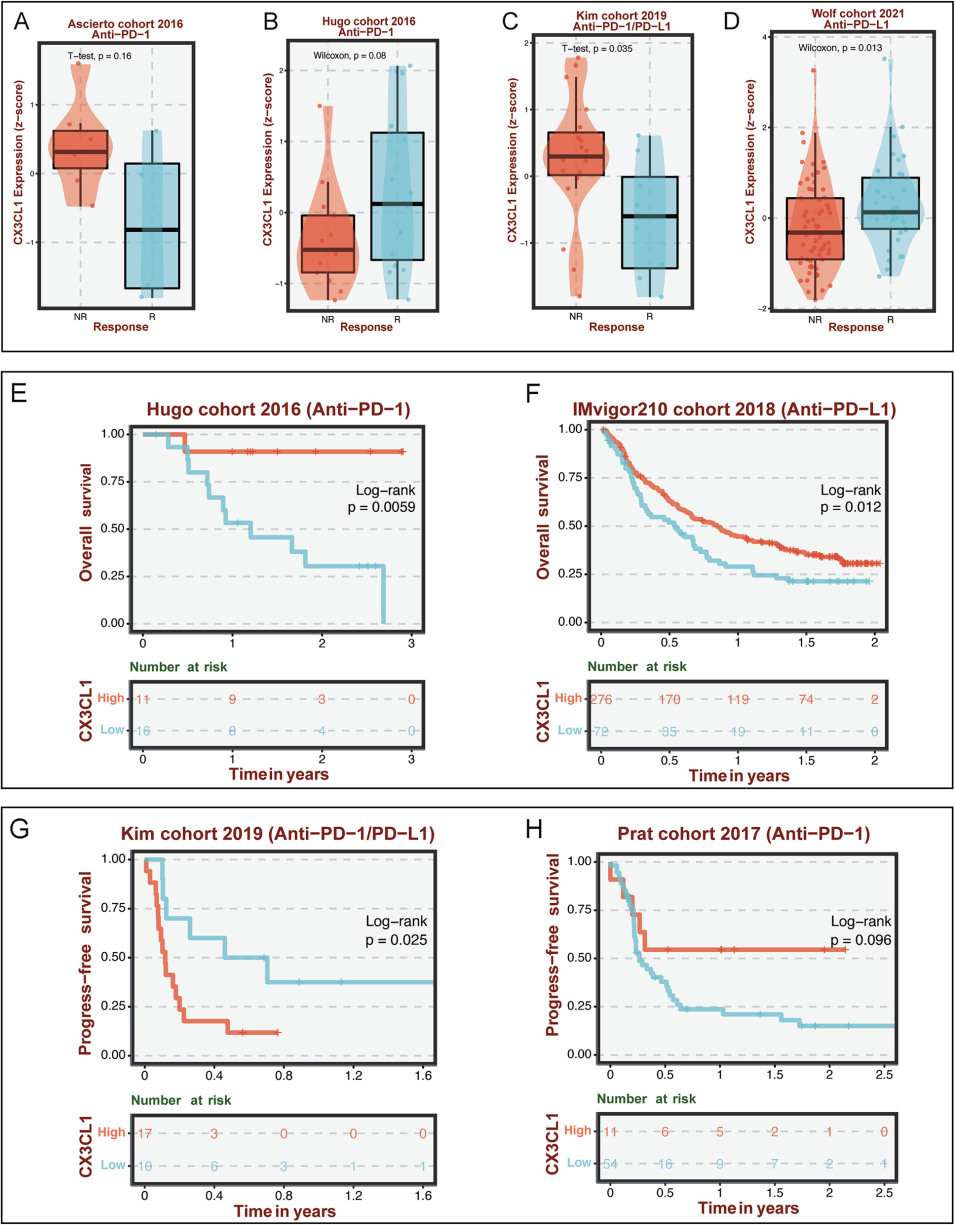
Immunotherapy analyses in different cohorts. **A-D** The relationship between the expression of CX3CL1 and the response to immunotherapy in the Ascierto, Hugo, Kim, and Wolf cohorts. **E-H** The relationship between the expression of CX3CL1 and prognosis values in Hugo, IMvigor, Kim, and Prat cohorts

Since the abovementioned results revealed a high correlation between CX3CL1 expression and immune cell infiltration in the TME in ccRCC, we investigated the potential role of CX3CL1 in human cancer immunotherapy next. As shown in Fig. 6A–D, we assessed the association between CX3CL1 expression and the immunotherapy response in different immunotherapy cohorts of patients. Patients treated with an anti-PD-1 or anti-PD-1/PD-L1 agent were included in the analysis. The results from the Kim cohort 2019 (*P* = 0.035) and Wolf cohort 2021 (*P* = 0.013) demonstrated that CX3CL1 expression could be used to predict the immunotherapy response of patients, as the expression levels of CX3CL1 were distinct among different patients. Next, we obtained the survival data of some patients who received immunotherapy. We found that CX3CL1 could effectively distinguish the prognoses of patients who received immunotherapy in the Hugo cohort 2016 (*P* = 0.0059), IMvigor210 cohort 2018 (*P* = 0.012), Kim cohort 2019 (*P* = 0.025), and Prat cohort 2017 (*P* = 0.096) (Fig. 6E–H).

### Restriction of 786-O cell migration and proliferation by CX3CL1 and its involvement in Ferroptosis

**Fig. 7:**
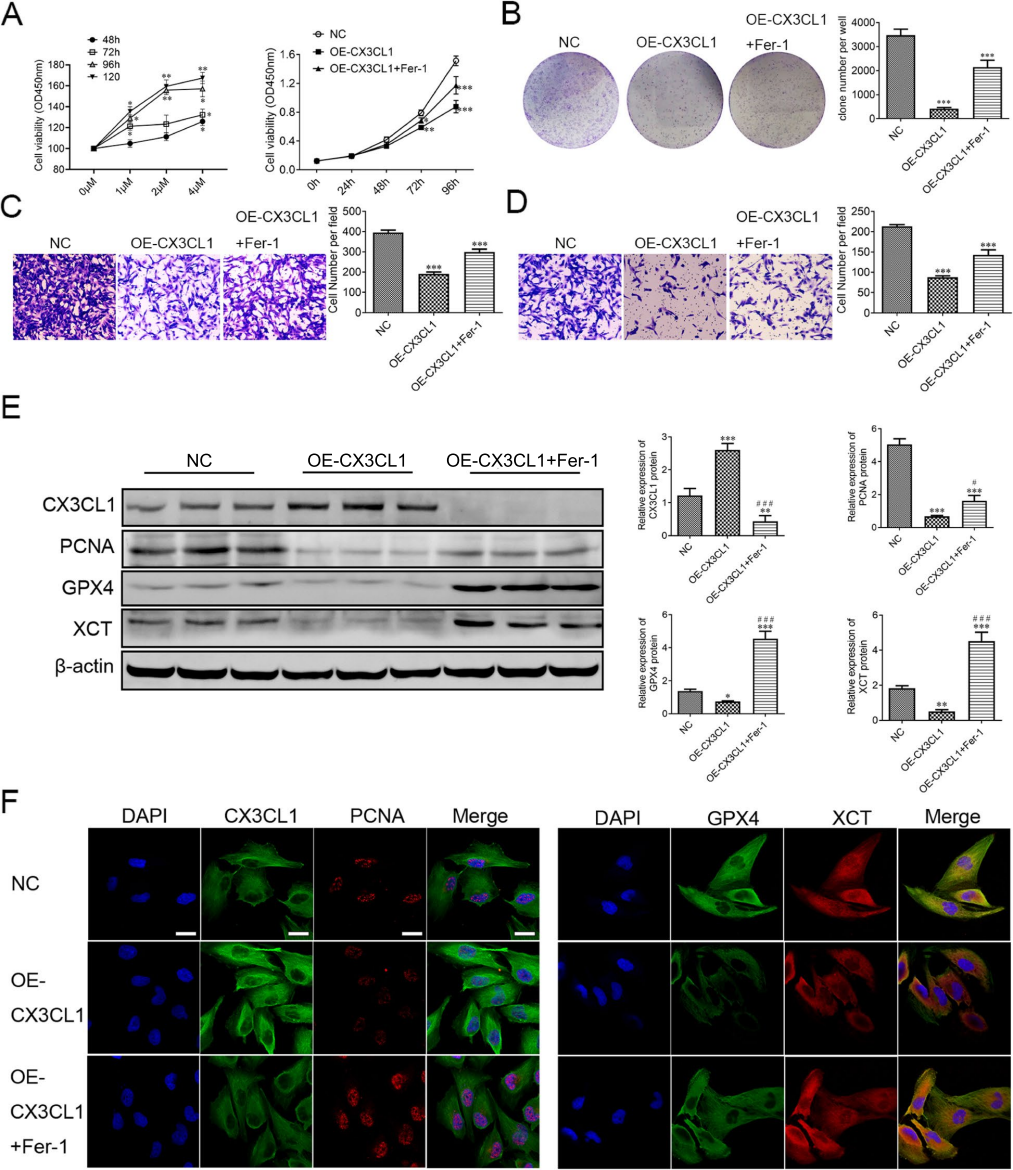
CX3CL1 inhibits ccRCC cell proliferation, migration, and invasion by inducing ferroptosis in *vitro*. **A** 786-O cells were incubated with Fer-1 (0, 1, 2, 4 μM/ml) for 48, 72, 96, and 120 h; then, cell viability was estimated by CCK-8 assay, and cell proliferation of 786-O cells in normal control, OE-CX3CL1, and OE-CX3CL1 + Fer-1 groups was estimated by CCK-8 assay. **B** Cell colonies of 786-O cells in normal control, OE-CX3CL1, and OE-CX3CL1 + Fer-1 groups. **C-D** 786-O cell migration and invasion assays were performed in normal control, OE-CX3CL1, and OE-CX3CL1 + Fer-1 groups. **E** Western blotting of CX3CL1, PCNA, GPX4, and XCT proteins. **F** The immunostaining assay identified the subcellular localizations of CX3CL1, PCNA, GPX4, and XCT. Scale bar: 10 μm. *P < 0.05, **P < 0.01, ***P < 0.001

Ferroptosis has been reported to play an important role in various cancers. Thus, we sought to elucidate the involvement of ferroptosis in ccRCC. To determine whether CX3CL1 induces ferroptosis in 786-O cells, we used Fer-1, a ferroptosis inhibitor, to investigate whether it can rescue 786-O cells from death caused by treatment with CX3CL1. We found that Fer-1 increased the viability of CX3CL1-treated 786-O cells in a concentration and time-dependent manner using the CCK-8 assay. At a 2 μM/ml concentration of Fer-1, the proliferation of 786-O cells was increased compared to that of normal cells after 96 h. In addition, our results revealed a significantly decreased cell proliferation in the CX3CL1-overexpressing group compared with that in the control group. Fer-1 treatment increased the proliferation ability of CX3CL1-overexpressing 786-O cells (Fig. 7A). Likewise, CX3CL1 overexpression significantly decreased the clone formation, cell migration, and invasion abilities in 786-O cells. Inversely, Fer-1 increased the clone formation, migration, and invasion abilities of CX3CL1-overexpressing 786-O cells (Fig. 7B–D). We also found a relationship between the inhibitory effects of CX3CL1 overexpression on 786-O cell proliferation and the effects of necroptosis, autophagy, and apoptosis inhibitors on 786-O cell proliferation. Necrostatin-1 (a necroptosis inhibitor), at 10 μM, increased 786-O cell proliferation compared to that in the control group 12 h after treatment (Supplementary Fig. 2A). Chloroquine (CQ, an autophagy inhibitor), at 25 μM, increased 786-O cell proliferation compared to the control group 24 h after treatment (Supplementary Fig. 2B). Z-VAD-FMK (an apoptosis inhibitor), at 20 μM, increased 786-O cell proliferation compared to the control group 48 h after treatment (Supplementary Fig. 2C). Clone formation analysis revealed that Fer-1 exerted a stronger effect on reversing the cell death caused by OE-CX3CL1 than necrostatin-1, CQ, and Z-VAD-FMK (Supplementary Fig. 2D). Further, PCNA (proliferating cell nuclear antigen) was found to be associated with the levels of 786-O cell proliferation. Western blotting for the protein expression levels of CX3CL1, PCNA, GPX4, and XCT indicated that the expression level of CX3CL1 in the OE-CX3CL1 group was increased compared with that in the control group, whereas the expression levels of PCNA, GPX4, and XCT were decreased; treatment with Fer-1 increased the expression levels of PCNA, GPX4, and XCT in the OE-CX3CL1 group (Fig. 7E, Supplementary Fig. 3). Immunofluorescence staining showed that CX3CL1 overexpression decreased the expression levels of GPX4 and XCT in the cytoplasm in 786-O cells. Fer-1 treatment reversed the effects of CX3CL1 overexpression on these proteins, as expected (Fig. 7F). These results indicate that CX3CL1 is vital in suppressing 786-O cell proliferation and invasion by promoting ferroptosis.

### Inhibition of tumor growth by CX3CL1 in *vivo*

**Fig. 8:**
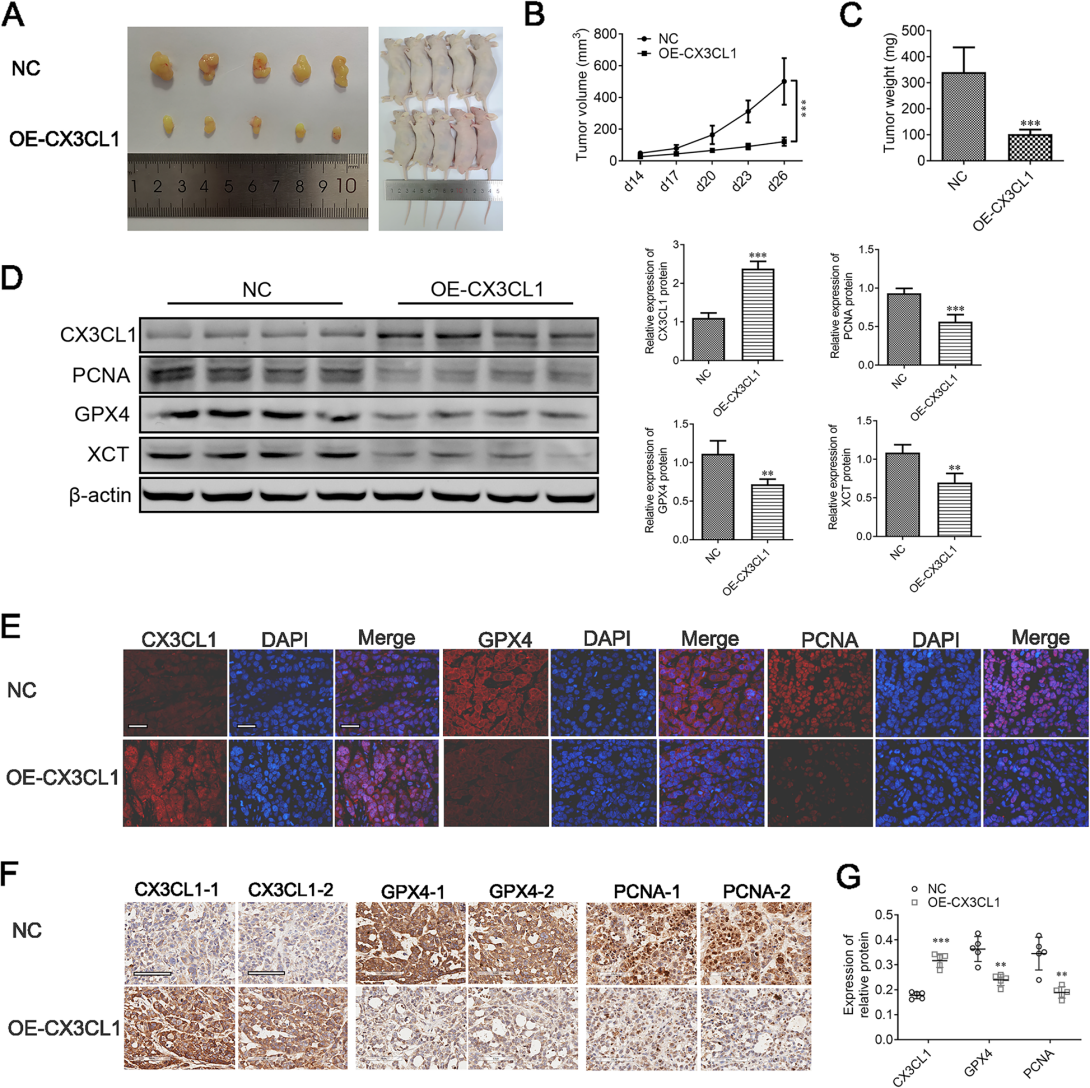
CX3CL1 inhibits ccRCC proliferation by inducing ferroptosis in *vivo*. **A** The representative images of xenografts in nude mice. **B–C** Tumor growth and weights of xenograft tumors from normal control and OE-CX3CL1 786-O cells. **D** Western blotting of CX3CL1, PCNA, GPX4, and XCT proteins. **E** The immunostaining assay identified the CX3CL1, GPX4, and PCNA proteins’ localization in xenografts tumors. **F-G** Representative images and quantitative analysis of CX3CL1, GPX4, and PCNA by IHC assay. Scale bar: 50 μm. *P < 0.05, **P < 0.01, ***P < 0.001

To determine the effects of CX3CL1 in *vivo*, we established a subcutaneous xenograft nude mouse model using 786-O cells. We found that the tumors in the OE-CX3CL1 group were significantly smaller and had a lower weight than those in the control group (Fig. 8A–C). Western blotting results showed that CX3CL1 overexpression decreased the expression of PCNA, GPX4, and XCT in OE-CX3CL1 group tissues compared with that in the control tissues (Fig. 8D, Supplementary Fig. 4). In addition, immunofluorescence staining using xenograft ccRCC tumor tissues showed increased localization of CX3CL1; GPX4 and PCNA were found to have decreased expression in the OE-CX3CL1 group compared to that in the control group (Fig. 8E). Furthermore, IHC staining showed that CX3CL1 was up-regulated while GPX4 and PCNA were down-regulated in the OE-CX3CL1 group compared with the control group (Fig. 8F–G). As we had expected, the results of our in *vivo* and in *vitro* tests were highly consistent. Thus, we reason that CX3CL1 overexpression can improve ccRCC prognosis by inducing ferroptosis-mediated tumor-cell death.

## Discussion

The most common type of malignant kidney tumor is renal cell carcinoma (RCC), with more than 350,000 people diagnosed with the disease each year worldwide [20]. Clear cell renal cell carcinoma (ccRCC) is the most common and aggressive pathological type of RCC and has high heterogeneity. Abundant vascularization in ccRCC tumors contributes to the proliferation of ccRCC cells, the ability of these cells to metastasize through blood flow, and the formation of a distinct tumor microenvironment (TME) [21]. Although immune checkpoint inhibitors (ICI) have improved ccRCC treatment, their lasting benefits are limited to a few patients. Most patients with ccRCC develop drug resistance to ICIs, resulting in a poor prognosis [22]. Therefore, it is crucial to reveal the biomarkers that regulate the development of ccRCC and identify novel treatment options. In this study, by exploring the expression of CX3CL1 pan-cancer and its association with prognosis, we determined that CX3CL1 is involved in the development of ccRCC and the survival of patients with ccRCC. Further analysis revealed that there is a potential relationship between CX3CL1 expression and immune cells in the TME and that CX3CL1 can thus influence cancer immunotherapy. We also found that CX3CL1 overexpression can inhibit the proliferation and migration of ccRCC cells and increase the sensitivity of ccRCC cells to ferroptosis. These findings provide a new perspective on the role of CX3CL1 in ccRCC and suggest the potential of a combined treatment strategy involving CX3CL1-mediated tumor immunotherapy and ferroptosis regulation in ccRCC.

Studies have controversially shown that CX3CL1 might either inhibit or promote tumorigenesis. For example, CX3CL1 as a chemokine has been associated with better patient outcomes by recruiting NK and CD8^+^ T cells in gastric adenocarcinoma [23]; paradoxically, CX3CL1 was also found to stimulate the proliferation of gastric and ovarian cancer cells by activating EGFR [24, 25]. Huang et al. found that JAK/STAT signaling was promoted by CX3CL1, leading to the growth of pancreatic ductal adenocarcinoma cells [26]. However, the function of CX3CL1 in ccRCC is still unclear. Our study found that CX3CL1 was abnormally expressed in ccRCC tissues compared with normal tissues. Moreover, CX3CL1 expression was differentially correlated with various tumor histologies and stages. Low CX3CL1 expression was associated with increased ccRCC malignancy and poor prognosis. These data suggest that a good prognosis in ccRCC linked to CX3CL1 may partly be based on the expression level of CX3CL1 as a tumor suppressor. Numerous reports suggest that CX3CL1 can effectively recruit NK cells and T cells into the TME and is a key promoter of anti-tumor activity [27–30]. Therefore, we believe that one of the mechanisms through which CX3CL1 affects patient prognosis in cancers is by influencing the host immune response.

Studies have shown that evolutionary pressure can influence tumorigenesis by acting on the cellular genome through a series of epigenetic mechanisms [31, 32]. As an essential part of the epigenetic mechanism, DNA methylation is involved in DNA repair, the cell cycle, and cell proliferation by regulating gene expression and signal transduction [33]. In this study, we found that DNA methylation is an epigenetic mechanism that negatively regulates CX3CL1 in ccRCC. Furthermore, with an increased expression of CX3CL1, its methylation levels were found to decrease, indicating that the methylation levels of CX3CL1 at various methylation sites are negatively correlated with ccRCC prognosis. The decrease of CX3CL1 expression in tumor cells is related to the chemical modification of its DNA by methylation; this mechanism is known as epigenetic silencing and may result from evolutionary pressure on the cellular genome. Recently, studies have found that specific DNA methylation sites could be valuable prognostic and diagnostic tools in cancer. Katzendorn et al. found that patients with high INA, NHLH2, and THBS4 gene methylations showed a worse survival rate in ccRCC than those with low methylations [34]. Yang et al. found that linc-ZNF582-AS1 hypermethylation was related to a shorter survival rate in ccRCC patients [35]. Our study precisely identified seven CX3CL1 methylation sites as ccRCC prognostic targets. Moreover, we found that the methylation site cg27664018 is a representative site among all seven sites, which can be a subject of future investigations. These sites can aid precision medicine for ccRCC.

According to immunophenotypes of tumor immune microenvironments of patients, tumors can be divided into inflamed, non-inflamed, and immune-excluded tumors [36]. Renal cell carcinoma, a typical immune-excluded tumor, is considered to lack the expression of some chemokines related to T cell recruitment, such as CXCL9, CXCL10, CXCL11, CXCL13, CX3CL1, CCL2, and CCL5, in its TME [37], but the specific mechanisms underlying this lack of chemokines remain unclear. CX3CL1 is considered an essential chemokine in regulating cancer progression. So far, various studies have provided conflicting results regarding the role of CX3CL1 in anti-tumor immunity. Recent studies have suggested that signaling through the ligand-receptor pair formed by CX3CL1 and its receptor CX3CR1 may improve the prognosis of ccRCC patients [38]. However, in another new clinical trial, the chemokine CX3CL1 was associated with shorter PFS in metastatic triple-negative breast cancer [39]. In the present study, CX3CL1 expression in ccRCC was significantly correlated with CD8^+^ T cell infiltration into the TME. Interestingly, previous studies believed that, generally, the infiltration of CD8^+^ T cells into the TME of solid tumors is associated with improved prognosis, but in ccRCC, this infiltration is associated with worse prognosis [40]. However, in some ccRCC patients with a “normal” tumor immune environment, the expression of perforin on CD8^+^ T cells with high infiltration levels was related to a better prognosis [41]. After receiving antigen stimulation, CD8^+^ T cells can differentiate into either effector CD8^+^ T cells or exhausted CD8^+^ T cells, depending on their different pathological states [42]. Exhausted CD8^+^ T cells can limit infection by some pathogens or the tumor immune response to reduce immune-mediated pathological damage, but the result of this restrictive function is often the continuous progress or deterioration of the disease [42]. In recent studies, it has been reported that, with the progression of renal cell carcinoma, the accumulation of exhausted CD8^+^ T cells leads to progressive immune dysfunction; this may be one of the reasons why CD8^+^ infiltrating T cells are associated with a worse prognosis in ccRCC [43]. However, studies have also reported that the level of infiltration of activated CD8^+^ T cells decreased significantly in advanced tumor samples [43, 44]. As the same, we speculate that activated CD8^+^ T cells are associated with CX3CL1 in this study. Nevertheless, our results generally show the uniqueness of the tumor immune microenvironment in ccRCC. At the same time, our results indicate that CX3CL1 can restore or enhance the anti-tumor activity of activated CD8^+^ T cells in the tumor immune microenvironment and, thereby, reshape the tumor immune microenvironment to improve the anti-tumor immunity of advanced ccRCC.

Because the tumor immune microenvironment plays a crucial role in cancer development and response to immunotherapy, many studies have used existing immunotherapy cohorts to evaluate the applicability of tumor immune microenvironment-related molecular markers in tumor immunotherapy [45, 46]. However, due to insufficient cohorts, most studies only use a small number of research cohorts for evaluation. It is worrisome that the different phenotypes of tumor cells, in terms of proliferation, cell morphology, and metastatic potential, cause tumor heterogeneity that may critically impact response to a particular treatment modality [47]. This makes using a single cohort to evaluate the potential of an immunotherapeutic agent limited in clinical application. In this study, as many immunotherapy cohorts as possible were collected to evaluate the value of CX3CL1 in predicting the immunotherapy response to it. The results showed that CX3CL1 had different effects on the response to immunotherapy in different tumors. For instance, in melanoma, high expression of CX3CL1 was associated with a better prognosis after immunotherapy. However, in non-small cell lung carcinoma, high expression of CX3CL1 was associated with a worse prognosis after immunotherapy. In general, these data confirm the potential ability of CX3CL1 in predicting the response to immunotherapy and show that CX3CL1 is a promising biomarker for cancer immunotherapy. Meanwhile, it suggests that concentrating on the differences in immunotherapy response caused by tumor heterogeneity will help us better understand the clinical applicability of CX3CL1 in ccRCC.

Although immunotherapy by PD-1/PD-L1 immune checkpoint blockade has achieved satisfying clinical results in a variety of solid tumors, including ccRCC, the low response rate of a single drug and the emergence of drug resistance have indicated the need for improving clinical benefits. Recent studies have reported that CD8^+^ T cell-mediated ferroptosis affects anti-tumor immunity induced by immunotherapy [48]. Previous results showed that CX3CL1 expression positively correlated with CD8^+^ T cell infiltration in ccRCC. It was suggested that CX3CL1 has the potential to induce ferroptosis in ccRCC. Moreover, as a form of non-apoptotic cell death, ferroptosis, characterized by iron-dependent lipid peroxide accumulation, has been identified as an effective target for kidney disease [49]. Thus, we further investigated the effect of CX3CL1 on ferroptosis in ccRCC cells in this study. We found that CX3CL1 inhibited ccRCC proliferation in *vivo* and in *vitro*. Fer-1 treatment decreased the anti-tumor effects of CX3CL1, demonstrating the critical role played by the ferroptosis regulation activity of CX3CL1 in its anti-tumor effect. Further mechanistic studies showed that overexpression of CX3CL1 leads to the suppression of GPX4 and XCT proteins and promotes the ferroptosis sensitivity of ccRCC cells. The GSH/GPX4 pathway is critical for regulating ferroptosis progression [50]. Inhibiting GPX4 by XCT suppression has been a practical way to induce cancer cell ferroptosis. As an essential ferroptosis regulator, GPX4 commits GSH to glutathione disulfide, resulting in reduced cholesterol hydroperoxide and oxidized fatty acid production, thereby preventing oxidation damage [51]. Concurrently, studies have found that the ferroptosis inducer Erastin is a promising cancer therapeutic modality since it could suppress intracellular GSH synthesis by mediating system Xc − [52]. In combination with the abovementioned findings of previous studies, our results show that CX3CL1 can promote ferroptosis sensitivity in tumor cells, but the complex relationship between CX3CL1, immune cell infiltration, and ferroptosis in ccRCC still needs more research for further clarification.

Overall, this study preliminarily characterized the potential role of CX3CL1 in the tumor immune microenvironment and in promoting the ferroptosis sensitivity of tumor cells. There are some limitations to our research. First, the clinical significance of CX3CL1 may need to be verified in an expanded clinical sample cohort. In addition, although we have used as many reliable algorithms and high-quality cohorts as possible to analyze the potential relationship between CX3CL1 and the TME of ccRCC, it is also necessary to determine the effect of CX3CL1 on T cell infiltration into the TME through more detailed experimental studies. Finally, the direct mechanism underlying CX3CL1 participation in ferroptosis in ccRCC in this study still needs further study for clarification.

In summary, our study demonstrated that the expression of CX3CL1 is associated with patient prognosis and may play a vital role in regulating the TME in ccRCC. Furthermore, to our knowledge, this is the first study describing the relationship between CX3CL1 and ferroptosis sensitivity in ccRCC. Targeting CX3CL1 may thus be a potential therapeutic strategy for patients with ccRCC. Exploring CX3CL1 further will improve our understanding of the combined effects of ferroptosis induction and immunotherapy on patient outcomes in ccRCC.

## Supplementary Information

**Additional file 1: Table 1.** Abbreviations of cancers in the TCGA-Pancancer cohort.

**Additional file 2: Supplementary Fig. 1.** The association between CX3CL1 expression and NK cells. (A–C) The relationship between activated NK cells and the expression level of CX3CL1 in GSE29069, E_MTAB_1980, and ICGC_EU cohorts determined using CIBERSORT. (D–F) The relationship between activated NK cells and the expression level of CX3CL1 in GSE29069, E_MTAB_1980, and ICGC_EU cohorts determined using CIBERSORT_ABS.

**Additional file 3: Supplementary Fig. 2.** The relationship between CX3CL1 and necroptosis, autophagy, and apoptosis. (A) 786-O cells were incubated with Necrostatin-1 (0, 5, 10, 15 μM/ml) for 6, 12, and 24 h. Then cell viability was estimated using the CCK-8 assay. (B) 786-O cells were incubated with CQ (0, 5, 15, 25 and 35 μM/ml) for 12, 24, and 48 h. Then cell viability was estimated using the CCK-8 assay. (C) 786-O cells were incubated with Z-VAD-FMK (0, 10, 20, and 30 μM/ml) for 24, 48, and 72 h. Then cell viability was estimated using the CCK-8 assay. (D) Cell colonies of 786-O cells.

**Additional file 4: Supplementary Fig. 3.** The full-length blots of Actin, CX3CL1, GPX4, PCNA and XCT in *virto*.

**Additional file 5: Supplementary Fig. 4.** The full-length blots of Actin, CX3CL1, GPX4, PCNA and XCT in vivo.

## Data Availability

Publicly available database analyzed in this study can be found in the The Cancer Gernome Altas (https://portal.gdc.cancer.gov/).
