# Supplementary material for: CX3CL1 promotes cell sensitivity to ferroptosis and is associated with the tumor microenvironment in clear cell renal cell carcinoma
Source: BMC Cancer. 2022 Nov 17;22:1184. doi: 10.1186/s12885-022-10302-2 (PMC9670481; doi:10.1186/s12885-022-10302-2)
Supplement: Supplementary file 2 — Additional file 2: Supplementary Fig. 1. The association between CX3CL1 expression and NK cells. (A–C) The relationship between activated NK cells and the expression level of CX3CL1 in GSE29069, E_MTAB_1980, and ICGC_EU cohorts determined using CIBERSORT. (D–F) The relationship between activated NK cells and the expression level of CX3CL1 in GSE29069, E_MTAB_1980, and ICGC_EU cohorts determined using CIBERSORT_ABS. [file 12885_2022_10302_MOESM2_ESM.pdf]

activated NK cells

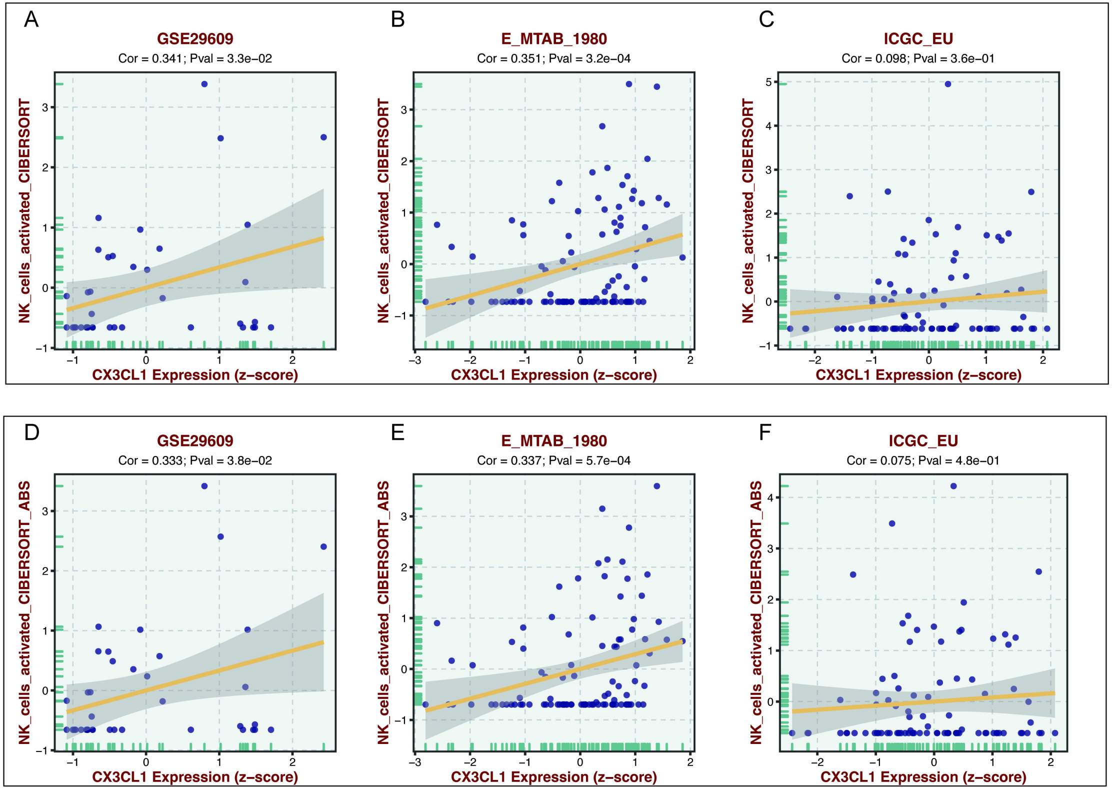

Supplementary Fig 1. The association between CX3CL1 expression and NK cells. (A–C) The relationship between activated NK cells and the expression level of CX3CL1 in GSE29069, E_MTAB_1980, and ICGC_EU cohorts determined using CIBERSORT. (D–F) The relationship between activated NK cells and the expression level of CX3CL1 in GSE29069, E_MTAB_1980, and ICGC_EU cohorts determined using CIBERSORT_ABS.
