# Supplementary material for: CX3CL1 promotes cell sensitivity to ferroptosis and is associated with the tumor microenvironment in clear cell renal cell carcinoma
Source: BMC Cancer. 2022 Nov 17;22:1184. doi: 10.1186/s12885-022-10302-2 (PMC9670481; doi:10.1186/s12885-022-10302-2)
Supplement: Supplementary file 3 — Additional file 3: Supplementary Fig. 2. The relationship between CX3CL1 and necroptosis, autophagy, and apoptosis. (A) 786-O cells were incubated with Necrostatin-1 (0, 5, 10, 15 μM/ml) for 6, 12, and 24 h. Then cell viability was estimated using the CCK-8 assay. (B) 786-O cells were incubated with CQ (0, 5, 15, 25 and 35 μM/ml) for 12, 24, and 48 h. Then cell viability was estimated using the CCK-8 assay. (C) 786-O cells were incubated with Z-VAD-FMK (0, 10, 20, and 30 μM/ml) for 24, 48, and 72 h. Then cell viability was estimated using the CCK-8 assay. (D) Cell colonies of 786-O cells. [file 12885_2022_10302_MOESM3_ESM.pdf]

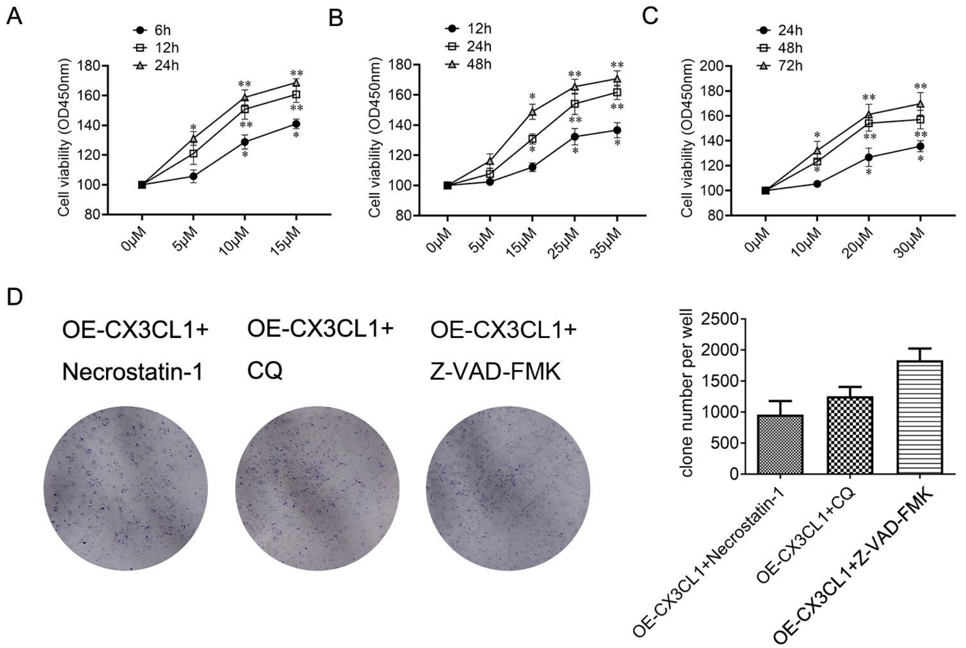

Supplementary Fig 2. The relationship between CX3CL1 and necroptosis, autophagy, and apoptosis. (A) 786-O cells were incubated with Necrostatin-1 (0, 5, 10, 15 μM/ml) for 6, 12, and 24 h. Then cell viability was estimated using the CCK-8 assay. (B) 786-O cells were incubated with CQ (0, 5, 15, 25 and 35 μM/ml) for 12, 24, and 48 h. Then cell viability was estimated using the CCK-8 assay. (C) 786-O cells were incubated with Z-VAD-FMK (0, 10, 20, and 30 μM/ml) for 24, 48, and 72 h. Then cell viability was estimated using the CCK-8 assay. (D) Cell colonies of 786-O cells.
