# Supplementary material for: CX3CL1 promotes cell sensitivity to ferroptosis and is associated with the tumor microenvironment in clear cell renal cell carcinoma
Source: BMC Cancer. 2022 Nov 17;22:1184. doi: 10.1186/s12885-022-10302-2 (PMC9670481; doi:10.1186/s12885-022-10302-2)
Supplement: Supplementary file 5 — Additional file 5: Supplementary Fig. 4. The full-length blots of Actin, CX3CL1, GPX4, PCNA and XCT in vivo. [file 12885_2022_10302_MOESM5_ESM.pdf]

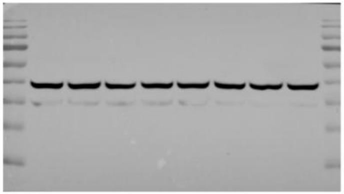

Actin

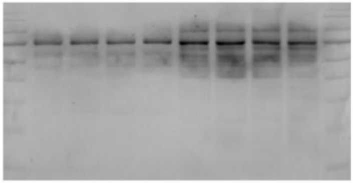

CX3CL1

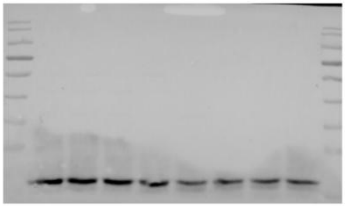

GPX4

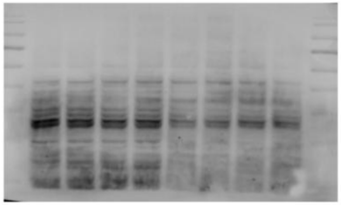

PCNA

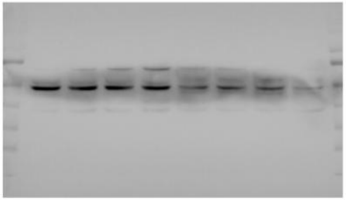

XCT

Supplementary Fig 4. The full-length blots of Actin, CX3CL1, GPX4, PCNA and XCT in *vivo*.
